# Promoting resilience and sexual and reproductive health among adolescent migrants: a comprehensive approach

**DOI:** 10.3389/fpsyt.2025.1719581

**Published:** 2026-02-09

**Authors:** Alexios Georgalis, Robert Thomson

**Affiliations:** 1 Department of Psychology, National and Kapodistrian University of Athens, Athens, Greece; 2 Department of Social Work and Psychology, University of Gävle, Gävle, Sweden

**Keywords:** adolescent resilience, Italy, life-skills education, migrant psychiatry, psychosocial support, sexual and reproductive health, trauma-informed care, unaccompanied minors

## Abstract

**Background:**

Migrant adolescents in Italy face intersecting risks that compromise mental health, including trauma exposure, legal precarity, and systemic barriers to care. Sexual and reproductive health (SRH) is a critical yet often overlooked determinant of adolescent wellbeing. Despite international guidelines endorsing adolescent SRH rights, such domains are rarely integrated into psychiatric care for displaced youth.

**Methods:**

We conducted a cross-sectional exploratory survey involving 58 migrant adolescents aged 15–17 years, all residing in Italy for less than five years. Data collection was facilitated by trained cultural mediators in Italian, Arabic, Bengali, French, and Urdu. The questionnaire assessed SRH knowledge, service awareness, gender attitudes, and self-reported resilience. Descriptive statistics were used to analyze item-level responses, supported by a narrative synthesis of resilience-oriented, life-skills-based interventions developed by UNFPA and UNICEF. Interpretation was grounded in trauma-informed and rights-based frameworks.

**Results:**

Findings revealed substantial SRH knowledge gaps, limited awareness of adolescent-friendly services, and frequent communication barriers with formal health providers. While patriarchal gender norms remained prevalent, attitudes showed signs of adaptation in longer-settled youth. Moderate-to-high resilience was observed across domains of self-efficacy, future orientation, and social connectedness. Notably, adolescents with greater SRH literacy reported higher mental health self-ratings and more confidence navigating local services.

**Conclusion:**

Integrating SRH literacy and culturally adapted life-skills education into migrant psychiatry offers a promising pathway to enhance adolescent resilience and reduce psychiatric vulnerability. Trauma-informed, participatory models—delivered through trusted mediators—can address both knowledge gaps and emotional distress, aligning psychiatric care with the complex lived realities of migrant youth in Italy.

## Introduction

Migration is a defining global force that shapes adolescent health and development. Among the more than 82 million displaced people worldwide, a significant proportion are adolescents aged 10–19 who face unique psychosocial challenges as they navigate identity formation in the context of displacement, trauma, and cultural transition (1–3). These adolescents include refugees, asylum seekers, unaccompanied minors, and children of labor migrants. While each subgroup carries distinct experiences, they also share common barriers: disrupted education, language obstacles, uncertainty about legal status, and exposure to structural and interpersonal discrimination (4–6). The cumulative impact of these barriers often manifests in mental health vulnerabilities, particularly anxiety, depression, and psychosocial withdrawal (7, 8). However, dominant psychiatric approaches tend to focus narrowly on trauma symptomatology, neglecting broader developmental needs—especially in areas like sexual and reproductive health (SRH). Yet SRH is a vital, identity-linked domain that shapes emotional wellbeing, self-worth, and relational competence during adolescence (9, 10).

## Toward a migrant psychiatry framework

Migrant psychiatry is increasingly recognized as a distinct and evolving subfield that challenges traditional clinical models by foregrounding the complex social, legal, and cultural determinants shaping migrant mental health. Unlike standard psychiatric frameworks that often rely on individualized diagnosis and treatment, migrant psychiatry interrogates the ways in which displacement, precarity, exclusion from health and legal systems, and institutional invisibility produce and perpetuate psychological suffering. Scholars have emphasized that psychiatric care for migrant populations should move beyond trauma-centric narratives to encompass rights-based, culturally competent, and structurally informed approaches (11–12). This includes attending to legal status, housing, access to SRH services, and intersecting vulnerabilities such as language exclusion, racism, and age-based power differentials. As such, migrant psychiatry draws from and contributes to fields such as cultural psychiatry, global health, medical anthropology, and migration studies, aligning itself with a broader movement toward socially responsive and ethically engaged mental health practice (13). The findings of this study underscore the need to consolidate migrant psychiatry as a field that does not merely react to crisis, but proactively scaffolds resilience, agency, and inclusion through interdisciplinary collaboration.

There is increasing recognition that psychiatry should evolve to meet the needs of migrant youth. A growing body of work calls for a shift toward “migrant psychiatry”: a practice rooted in trauma-informed care, resilience theory, and rights-based youth development (14–16). This approach moves beyond diagnosing distress to understanding how migration, identity, gender norms, and systemic exclusion shape young people’s emotional lives. Within this framework, SRH becomes central—not peripheral—to adolescent mental health. Misinformation about contraception, fear of pregnancy, shame around sexuality, and lack of access to SRH services can generate profound anxiety, confusion, and depressive symptoms, particularly when compounded by isolation or conflicting cultural expectations (17–19). For migrant adolescents, these challenges are intensified by legal and linguistic barriers, taboos around sexuality, and exclusion from formal health education (20).

## The Italian context

Italy provides a particularly relevant setting to explore these issues. As a key point of arrival in Europe, Italy has received some of the highest numbers of unaccompanied migrant minors in the European Union. In 2022 alone, 14,044 unaccompanied minors arrived by sea, with adolescents aged 15 to 17 forming the largest group. By October 2023, another 11,592 unaccompanied minors had arrived, primarily from Egypt, Tunisia, Guinea, Côte d’Ivoire, Bangladesh, Pakistan, and Syria (21, 22). Despite Italy’s international commitments to adolescent rights and health, it lacks a national policy on comprehensive sexuality education (CSE). CSE remains optional, inconsistently implemented across regions, and largely unavailable in reception settings.

Migrant adolescents often rely on peer networks or unverified online platforms for information about puberty, contraception, or legal rights—exacerbating confusion, stigma, and psychological stress. Although Italy has piloted educational models such as the Boys on the Move curriculum, developed by UNFPA and UNICEF, these programs remain limited in scale, time-bound, and disconnected from mental health service frameworks (23–27). As a result, SRH remains a neglected yet central determinant of adolescent mental wellbeing—particularly in reception systems where therapeutic engagement rarely extends to sexuality, gender identity, or reproductive autonomy.

## Theorizing adolescence beyond age brackets

Adolescence is often defined chronologically, but age alone cannot capture the complexity of this life stage. It is a time of profound transformation—biological, cognitive, emotional, and social—shaped not only by internal maturation but also by external forces such as family, education, culture, and politics. While many global frameworks position adolescence as a period between dependency and autonomy, lived experiences often deviate from this trajectory, especially in contexts marked by instability, conflict, or migration. For some young people, adolescence may be truncated by adult responsibilities, while for others, it may be prolonged due to legal or structural constraints.

Rather than viewing adolescence as a uniform or linear phase, it is more accurate to see it as a transition marked by negotiation—of identity, relationships, roles, and belonging. Migrant adolescents often experience this negotiation under conditions of displacement, systemic exclusion, and socio-legal ambiguity. Their development is influenced as much by their journeys as by their environments. Understanding adolescence in this light allows psychiatry and allied fields to respond with greater sensitivity, flexibility, and cultural relevance.

## Adolescents as vulnerable—and resilient

Adolescence is a developmental window marked by identity formation, autonomy, and affective exploration. Migrant adolescents often undertake this journey in the context of forced mobility, cultural dislocation, and uncertainty. Predictably, they show higher rates of post-traumatic stress disorder (PTSD), depression, and internalizing symptoms than non-migrant peers (28–30). Yet not all outcomes are negative. Research highlights the protective role of school connection, social support, and adult mentorship, all of which enhance resilience even under high stress (31–33).

### Sexual and reproductive health as a psychiatric concern

Sexual and reproductive health (SRH) has historically been the domain of public health and reproductive medicine, often sidelined in psychiatric care. However, emerging research illustrates the psychiatric implications of SRH literacy, gender norms, and service access among adolescents. Studies show that better SRH knowledge and equitable gender attitudes correlate with improved mental wellbeing, including greater self-esteem, reduced anxiety, and enhanced emotional regulation (33, 34).

For migrant adolescents, this linkage is particularly relevant. SRH confusion is common due to interrupted education, language barriers, and taboos inherited from both origin and host cultures (35, 36). These barriers make it difficult for youth to access reliable information or confidential care. Fear of parental disapproval, shame around sexual development, and uncertainty about rights contribute to chronic stress, social withdrawal, and depressive symptoms (37, 38).

Moreover, SRH needs often remain invisible in migrant reception systems, which tend to prioritize physical health and administrative status over psychosocial development. Yet adolescents’ emotional lives are deeply tied to their experiences of bodily autonomy, sexual identity, and gender expectations. When left unaddressed, these domains exacerbate vulnerability to psychiatric distress, especially among unaccompanied minors and youth from patriarchal communities (9, 18, 39). Psychiatry should evolve to meet this need. A rights-based and trauma-informed psychiatric framework should consider SRH literacy and access not as peripheral, but as central to adolescent mental health. Programs that link psychosocial support with SRH education—delivered through trusted, culturally fluent mediators—are well-positioned to close this gap. Integration of SRH into migrant mental health services could serve as both preventive and therapeutic, especially for adolescents navigating identity, trauma, and transition (40, 41).

## Rationale and aims of the study

This study builds on emerging interdisciplinary initiatives by systematically examining the knowledge, beliefs, and resilience assets of migrant adolescents in Italy in relation to sexual and reproductive health (SRH). Our rationale is threefold:

First, migrant adolescents remain a critically understudied population in European psychiatry, despite elevated exposure to displacement-related stressors and barriers to care (41). While research has documented mental health risks among unaccompanied minors and asylum-seeking youth, few studies address SRH literacy or gendered psychosocial development as psychiatric concerns (42, 43).

Second, SRH literacy and access are essential—but frequently overlooked—determinants of adolescent mental health. SRH insecurity contributes to anxiety, shame, and isolation, especially when compounded by disrupted education, cultural taboos, and legal uncertainty (44).

Third, resilience theory and trauma-informed care provide robust frameworks for intervention. Both emphasize relational safety, cultural grounding, and empowerment, and have been shown to enhance outcomes in adolescent migrants when adapted to local contexts (45–47). Cultural mediators, in particular, play a unique role in bridging clinical goals with the lived realities of displaced youth (48).

By generating empirical data through a cross-sectional, culturally mediated survey, this study contributes to the emerging paradigm of migrant psychiatry—a practice model that integrates trauma-informed, rights-based, and resilience-oriented approaches. Findings aim to inform not only psychiatric theory but also public health, youth work, and policy reform.

Our research questions were:

- What are the SRH knowledge levels and service awareness of migrant adolescents in Italy?
- What attitudes and beliefs shape their SRH practices?
- How do resilience factors interact with SRH understanding and perceived mental wellbeing?

## Structure of the manuscript

This manuscript follows the IMRAD format:

- Methods: Details the cross-sectional survey, sampling, tools, and ethical safeguards.
- Results: Presents data on SRH knowledge, gender norms, service access, and resilience indicators.
- Discussion: Interprets the findings through the lenses of trauma, resilience, and cultural mediation, linking them to comparative European and global literature.
- Conclusion: Reflects on implications for psychiatry, health equity, and youth-focused policy—arguing for a new culture of care within migrant mental health systems.

In summary, this study situates migrant adolescents as active, adaptive agents navigating layered adversity. By connecting SRH, resilience, and psychiatry within a migration context, it challenges fragmented care models and proposes a holistic, interdisciplinary vision for adolescent mental health (49–51).

## Materials and methods

### Study design and rationale

This study employed a cross-sectional exploratory design to investigate sexual and reproductive health (SRH) knowledge, beliefs, attitudes, and indicators of resilience among migrant adolescents in Italy. Given the limited research on this population and the practical constraints inherent in engaging hard-to-reach adolescent migrant groups, an exploratory methodology was adopted. This approach prioritized cultural appropriateness, ethical feasibility, and the timely generation of actionable insights over representativeness or hypothesis testing (52, 53).

Unlike hypothesis-driven epidemiological studies, exploratory surveys are particularly suited to under-researched contexts in which the primary goal is not causal inference but rather the identification of patterns and service gaps that can inform program design and policy (8). In this context, the research was developed to align scientific rigor with the realities of migrant reception systems in Italy, where institutional access is often fragmented and adolescents are in transition.

### Setting and context

Data collection took place between 2019 and 2020 across three Italian cities—Rome, Palermo, and Catania—representing urban hubs with substantial migrant reception infrastructures. The study was implemented in collaboration with UNICEF and UNFPA offices and was embedded in broader SRH and psychosocial support efforts for adolescents. Italy serves as one of Europe’s primary entry points for unaccompanied and separated migrant children, receiving nearly 10,000 unaccompanied minors by sea in 2019 alone (4).

However, the Italian reception system remains highly variable in quality and scope. While some municipalities and NGOs provide integrated youth health and protection services, others operate under resource constraints with fragmented coordination. These disparities significantly affect young people’s access to healthcare, education, and reliable information, particularly in domains like SRH where stigma and silence are common.

### Participants and recruitment

A total of 58 adolescents participated in the study. Inclusion criteria were:

Aged between 14 and 19 years at the time of data collection;

Identifying as a migrant, refugee, asylum seeker, or unaccompanied minor;

Residing in Italy for less than five years

Providing informed assent or consent (and guardian consent when required).

Participants were recruited through cultural mediators and NGO networks with pre-existing relationships of trust. Recruitment was purposive to capture diversity in gender, language, and country of origin. The final sample was skewed male, reflecting broader arrival trends to Italy during the data collection period (54).

### Country of origin

The adolescents represented a wide geographic spread across West Africa, South Asia, and the Middle East. Country-of-origin data are as follows:

Nigeria (n=7; 12.1%)

Italy (n=7; 12.1%)*

Côte d’Ivoire (n=6; 10.3%)

Ghana (n=5; 8.6%)

Guinea (n=5; 8.6%)

Senegal (n=5; 8.6%)

Bangladesh (n=4; 6.9%)

Côte d’Ivoire (n=4; 6.9%)

Mali (n=4; 6.9%)

Pakistan (n=3; 5.2%)

Gambia (n=2; 3.4%)

Albania, Burkina Faso, India, Libya, Togo, Benin (n=1 each; 1.7%)

*Note: These Italian-born participants self-identified as second-generation migrants or asylum applicants returned under family reunification policies.

### Survey instrument

The survey was adapted from two validated instruments:

The UNICEF/UNFPA “12 Questions and Answers on SRHR” guide, designed to probe knowledge gaps and provide adolescent-friendly explanations across topics such as menstruation, contraception, Sexually Transmitted Infections (STI’s), and legal entitlements (55).

The Boys on the Move (BotM) curriculum, developed by UNFPA and UNICEF, which integrates life skills, psychosocial coping, and SRH education for adolescent migrants (55). Survey domains included: contraception knowledge (e.g., male condoms, emergency contraception, IUDs); pregnancy beliefs (e.g., fertility myths such as risk during menstruation); abortion legality and perceived accessibility; SRH and mental health service awareness; endorsement of gender norms and rights; sources of information (e.g., peers, pornography, social media); and language preferences for health content. Instruments were translated into Arabic, French, and Italian, with back-translation performed to ensure accuracy. English was also available. A pilot with 10 adolescents tested clarity and cultural resonance, resulting in minor modifications to language and layout.

### Data collection procedures

Surveys were administered in small group sessions (8–12 participants) or one-on-one, based on participant literacy and comfort. Sessions lasted 45–60 minutes and were facilitated by trained enumerators with support from cultural mediators. Confidentiality was emphasized, and all responses were self-recorded anonymously into sealed envelopes.

Participants were informed that they could skip any question and withdraw at any time. Any participant expressing emotional distress or urgent SRH concerns was referred to on-site psychosocial or medical staff. These referral pathways were pre-established with partner NGOs.

### Sample size considerations

The sample size of 58 was determined by logistical feasibility, ethical constraints, and access limitations in the field. No formal power calculation was used. Rather, the aim was to gather indicative data for pattern mapping, consistent with practice-based evidence methodologies in humanitarian and adolescent health research (56, 57).

### Data analysis

Survey data were entered into SPSS v27 and analyzed descriptively. Frequencies and percentages were calculated for categorical variables. Data were visualized in tables and graphs. Given the small sample size, no inferential statistics were conducted. Instead, findings are discussed in relation to relevant literature and service frameworks. This approach aligns with methodological guidelines for exploratory studies in humanitarian contexts, where the aim is to identify service needs and inform interventions, not to test hypotheses (58).

### Ethical considerations

Ethical clearance was granted by the UNICEF Italy Ethics Committee, in line with the Declaration of Helsinki and the UNICEF ERIC (Ethical Research Involving Children) guidelines on research involving children (59). Written informed consent was obtained from all participants. For minors, parental or guardian consent was obtained unless the adolescent was under state care as an unaccompanied minor, in which case institutional approval was provided. Sensitive questions were phrased in age-appropriate, non-judgmental language. Cultural mediators played a central role in ensuring participant comfort, clarity, and understanding. Participants were also given multilingual referral sheets listing local SRH and mental health services.

### Methodological rigor

Multiple measures were taken to enhance rigor, including triangulation of instruments, translation and back-translation, pilot testing, and use of cultural mediators. Field staff maintained reflexive field notes, capturing contextual observations, dynamics of trust, and ethical concerns during data collection. This reflexive stance helped contextualize findings and ensured alignment with the study’s trauma-informed, resilience-oriented framework.

### Researcher positionality and engagement with the field

The authors have been actively engaged in the fields of adolescent mental health, sexual and reproductive health (SRH), anti-trafficking and youth-focused psychosocial programming. Between 2016 and 2023, the authors collaborated as International Consultants with United Nations agencies including UNFPA and UNICEF on SRH education, life-skills curricula and trauma-informed care for adolescents in migratory and humanitarian contexts. Our work spanned multiple settings—including Greece, Bosnia and Herzegovina, Serbia, Italy and Nigeria—and involved co-developing and implementing the “Boys on the Move” curriculum in reception centers and youth shelters across Southern Europe. We have maintained ongoing collaborations with governmental bodies, NGOs, and adolescent-serving institutions in Italy, particularly in Rome, Palermo, and Catania. This sustained engagement enabled culturally responsive survey design, ethical sensitivity, and trust-building with local partners and participants. Our dual roles as practitioners and researchers contributed to a grounded, interdisciplinary approach that informed both the conceptual framing and fieldwork of the present study.

Due to the sensitive nature of the research context, including ongoing anti-trafficking interventions, and the need to ensure participant anonymity and institutional data protection compliance, publication was intentionally delayed until appropriate safeguards were fully in place.

## Results

This study surveyed 58 migrant adolescents between the ages of 14 and 19, residing in reception centers and shelters in Italy, to assess their knowledge, beliefs, and attitudes toward sexual and reproductive health (SRH), along with indicators of resilience and service awareness. The diverse sample—drawn from 17 countries including Nigeria (12.1%), Italy (12.1%), Côte d’Ivoire (10.3%), Ghana (8.6%), Guinea (8.6%), and others—represents a realistic cross-section of unaccompanied and recently arrived youth in Italian reception systems. Findings are presented across eight core domains: (1) contraceptive knowledge; (2) beliefs about pregnancy risk during menstruation; (3) perceptions of abortion legality; (4) awareness of SRH services; (5) attitudes toward sexual relationships; (6) sources of SRH knowledge and pornography; (7) information access regarding puberty/menstruation; and (8) language preferences.

### Contraceptive knowledge

“I’ve heard of condoms, but I don’t know how they work. In my country, no one explains these things. Here, it’s also hard because I don’t know where to ask.” — Male, 15, Nigeria.

**Figure 1:**
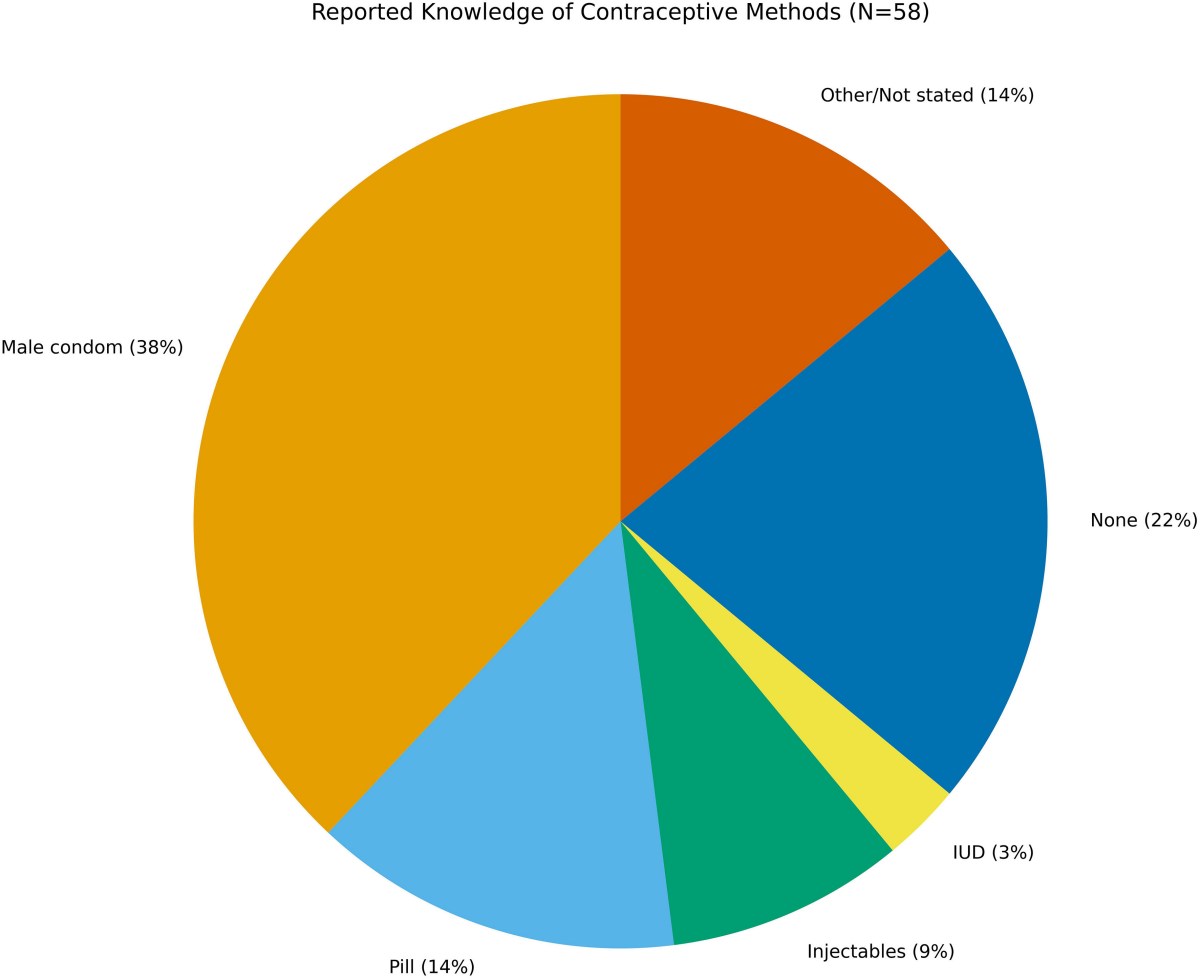
Knowledge of contraceptive methods among migrant adolescents (N = 58). Male condoms were the most frequently cited method (38%) followed by the pill (14%) injectables (9%) and intrauterine device (3%) while 22% knew no method at all. These findings illustrate significant gaps in contraceptive literacy among migrant adolescents in Italy.

Knowledge of contraceptive methods was inconsistent and overall limited (See **Figure 1**). Male condoms were the most frequently known method (38%, n=22). Far fewer adolescents could identify oral contraceptives (14%), injectables (9%), or intrauterine devices (IUDs) (3%). Alarmingly, 22% of respondents reported no knowledge of any contraceptive method. These findings echo patterns in both migrant and native-born adolescents across Europe, where contraceptive education is uneven and often excludes long-acting reversible contraception (LARC) (52, 53). The limited awareness of female-controlled or provider-dependent methods, such as IUDs or injectables, underscores the risk of relying solely on male-initiated contraception, particularly in contexts where communication about sex is restricted or influenced by gendered power dynamics.

### Fertility misconceptions: pregnancy during menstruation

**Figure 2:**
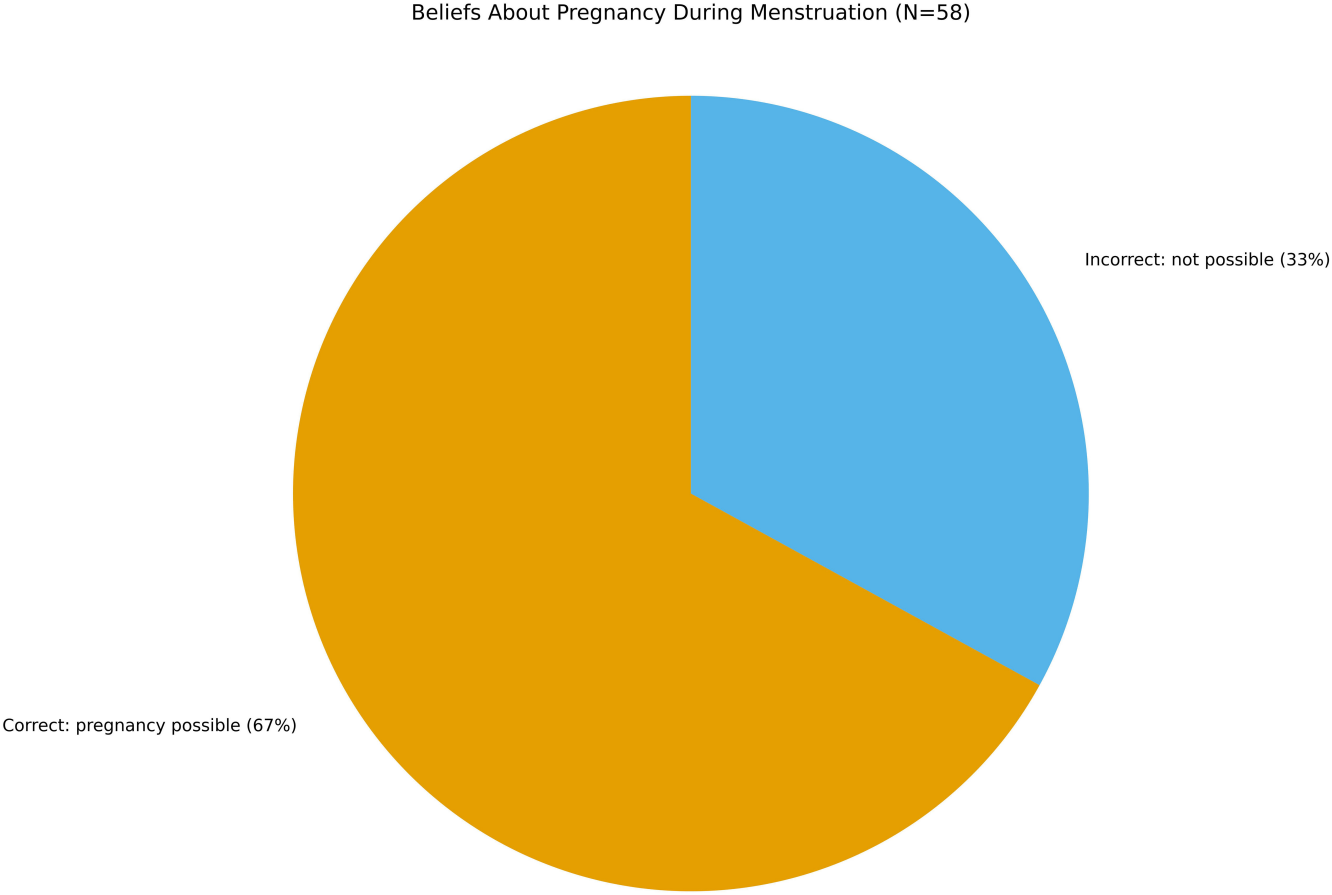
Beliefs about pregnancy during menstruation (N= 58) one third of respondents incorrectly believed pregnancy is not possible during menstruation, one-third were uncertain and one-third correctly recognized the possibility. Misconceptions about fertility reflect widespread myths observed in global adolescent populations.

One-third of participants erroneously believed that pregnancy is not possible during menstruation. (See **Figure 2**) Another third expressed uncertainty, while only 33% correctly recognized the possibility of conception during this phase of the menstrual cycle. This widespread misunderstanding aligns with global data on fertility myths among adolescents (8). Fertility misinformation can lead to unprotected sex, misinterpretation of bodily signals, and false perceptions of risk. For young migrants, whose educational trajectories have often been disrupted and whose exposure to biology curricula is fragmented, these misconceptions carry added weight.

When such beliefs intersect with gender-based taboos or shame, adolescents may avoid asking clarifying questions or seeking medical advice, instead relying on inaccurate peer information or online forums. From a clinical standpoint, these cognitive distortions can exacerbate worry and reproductive coercion, especially in relationships where one partner assumes control over decisions. Many adolescents in our sample demonstrated uncertainty or incorrect beliefs regarding the possibility of becoming pregnant during menstruation. This reflects broader trends identified in global research on fertility myths—widespread misconceptions about when and how conception can occur. Common myths include beliefs that pregnancy cannot happen during a girl’s first sexual encounter, that withdrawal is a reliable method, or that pregnancy is impossible during menstruation. Such misunderstandings contribute to inconsistent contraceptive use and unplanned pregnancies, particularly in contexts where comprehensive sexuality education is absent or inaccessible.

### Legal awareness: abortion in Italy

“Someone told me abortion is illegal here, like a crime. I don’t know what is true. I would be scared to ask a doctor.” — Female, 17, Ghana.

**Figure 3:**
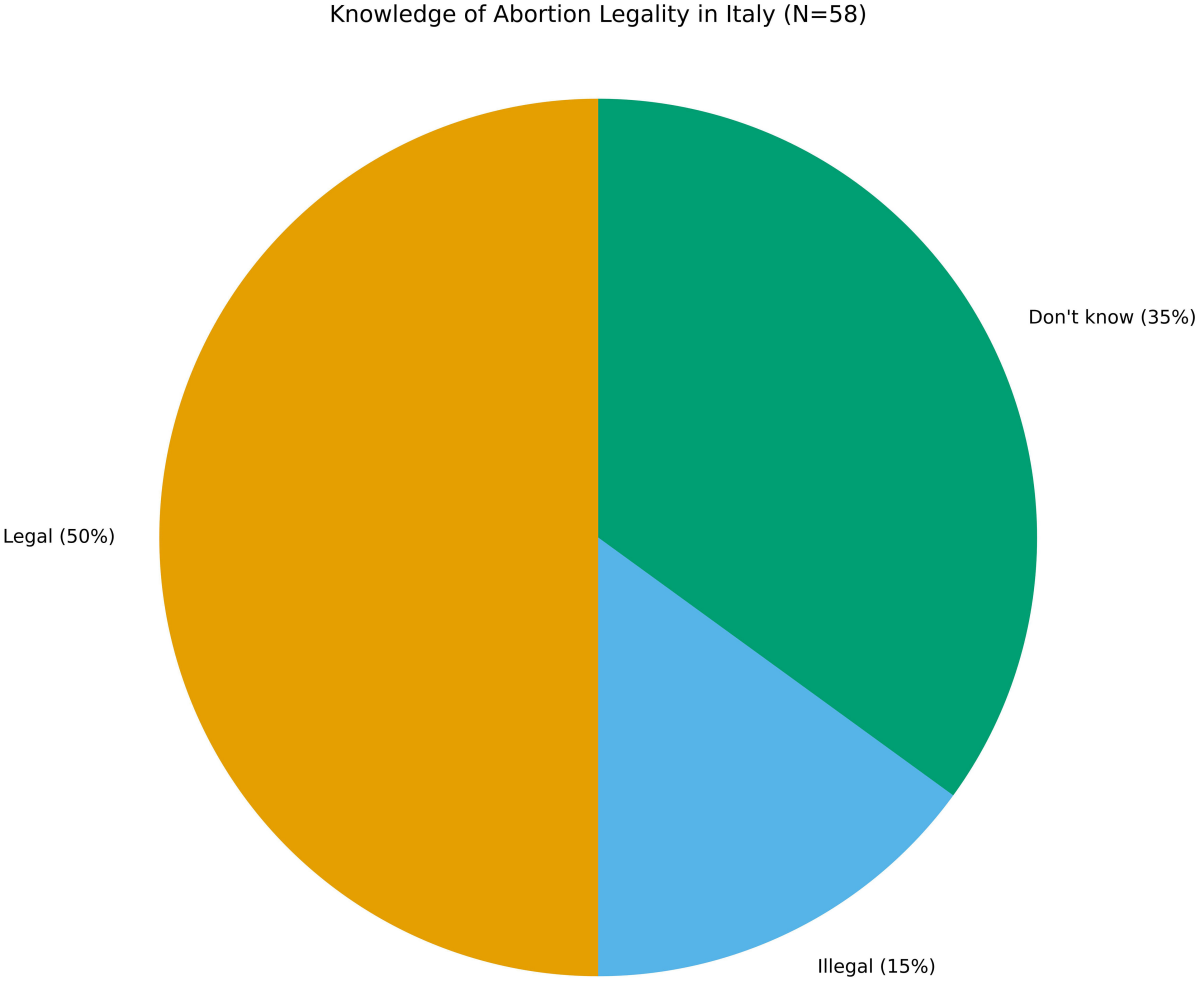
Knowledge of abortion legality in Italy (N=58). Half of respondents (50%) correctly identified abortion as legal while 15% stated it was illegal and 35% were unsure, indicating persistent confusion despite established national legislation.

Half of the adolescents (50%) were aware that abortion is legal in Italy, while 15% believed it was not, and 35% were unsure (See **Figure 3**). This uncertainty is concerning, particularly because abortion in Italy has been legal under certain conditions since 1978 (Law 194/78). Yet, the persistent stigma and uneven access to services—especially for migrants—undermine public awareness. Adolescents unfamiliar with their legal rights are less likely to seek appropriate medical care when facing an unintended pregnancy. Furthermore, the confusion around legality may contribute to psychological stress, delay in decision-making, and reliance on unsafe alternatives (4).

### SRH service awareness

**Figure 4:**
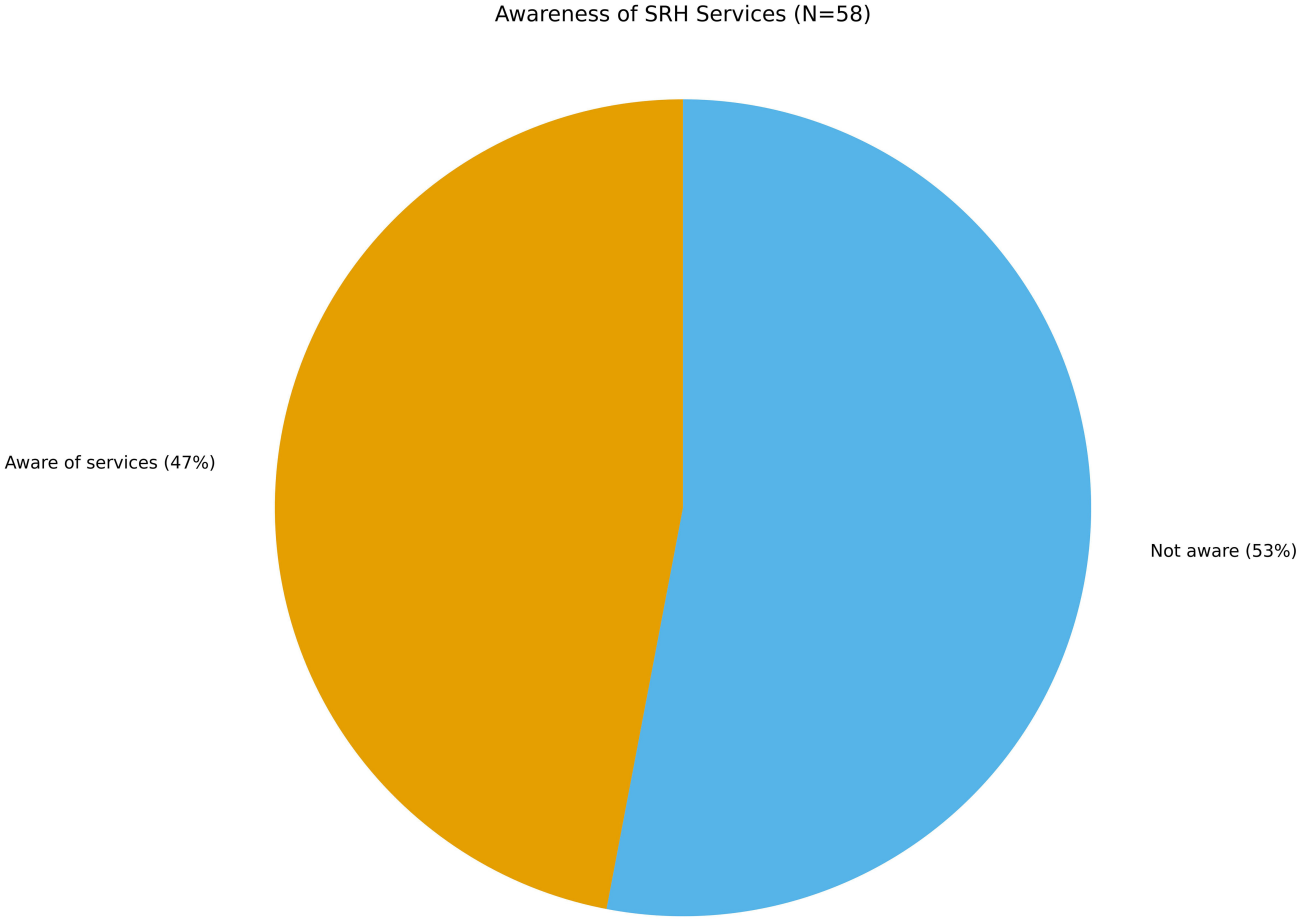
Awareness of sexual and reproductive health (SRH) services (N=58). Less than half (47%) reported knowing where to access SRH services, 33% said did not know and 21% were unsure. Awareness of services is a key determinant of healthcare utilization and protection behaviors.

Only 47% of participants reported knowing where to access SRH services. One-third stated they did not know, and 21% were unsure. These numbers are troubling given that awareness is a prerequisite for service use (See **Figure 4**). In Italy, despite the legal availability of youth-focused health services in some regions, adolescents—especially migrants—encounter fragmented, language-limited, and underfunded systems. Language barriers, unclear eligibility criteria, and fear of stigma act as powerful deterrents (54).

### Sexual attitudes and gender norms

“I think both boy and girl should feel good in a relationship. It’s about respect. Not just one person’s pleasure.” — Male, 17, Guinea.

**Figure 5:**
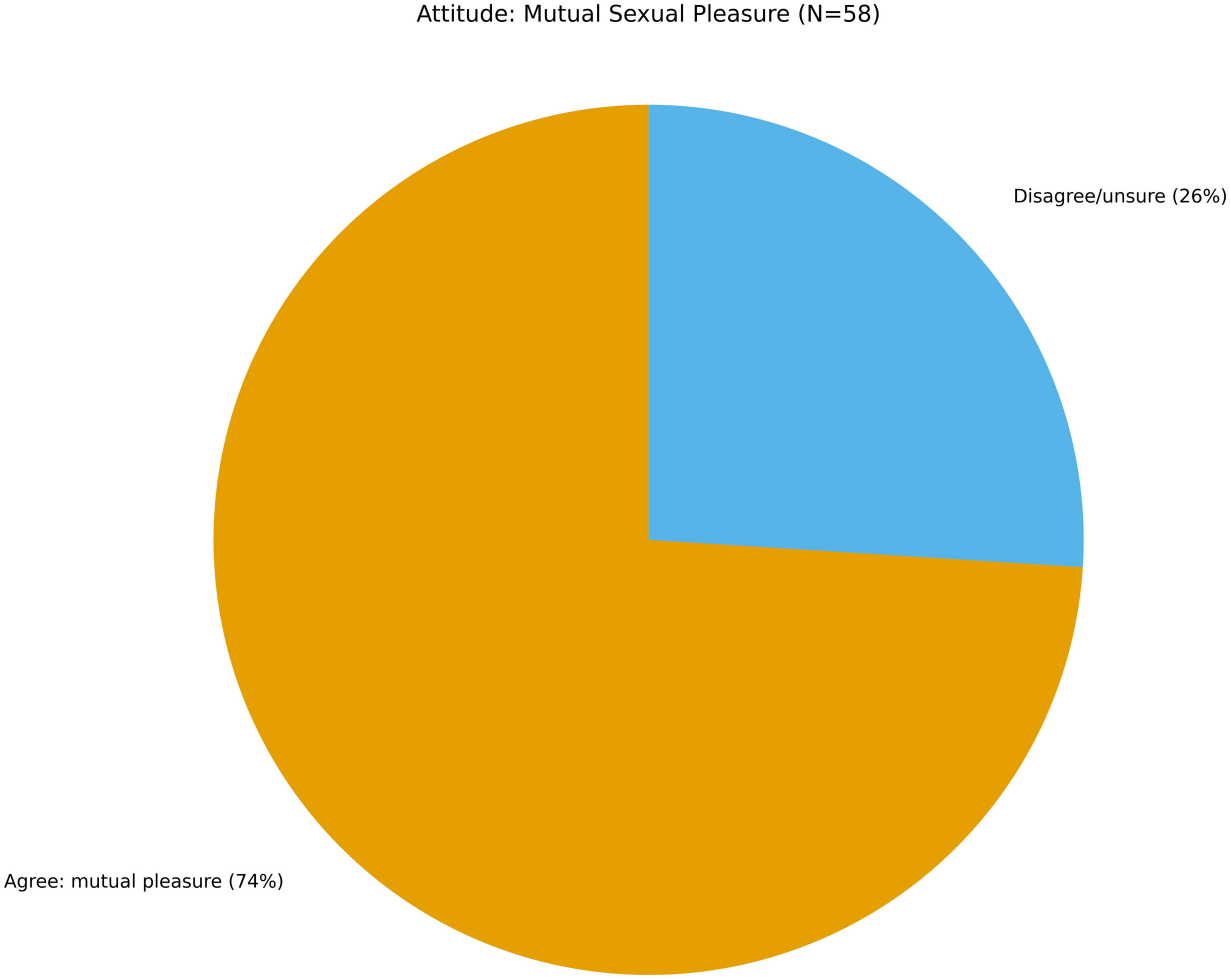
Attitude towards sexual relationship (N=58). A majority (74%) agreed that both partners should enjoy sexual relationships, indicating relatively progressive gender attitudes and highlighting a psychosocial resilience factor linked to quality and mutual respect.

Encouragingly, 74% of adolescents agreed that both partners should experience pleasure in sexual relationships (see **Figure 5**). This finding suggests a meaningful endorsement of gender equity in intimate relationships, which contrasts with traditional norms that may prioritize male pleasure or control. Positive sexual attitudes like this one are not merely ideological—they are linked to enhanced communication, reduced coercion, and more consistent contraceptive use (55). When adolescents believe that sexual satisfaction should be mutual, they are more likely to advocate for their own needs and respect those of partners.

### Sources of SRH knowledge and pornography exposure

“I learn from videos online. No one teaches us real things. At least in videos, you see what happens—even if it’s not always good.” — Male, 15, Côte d’Ivoire.

**Figure 6:**
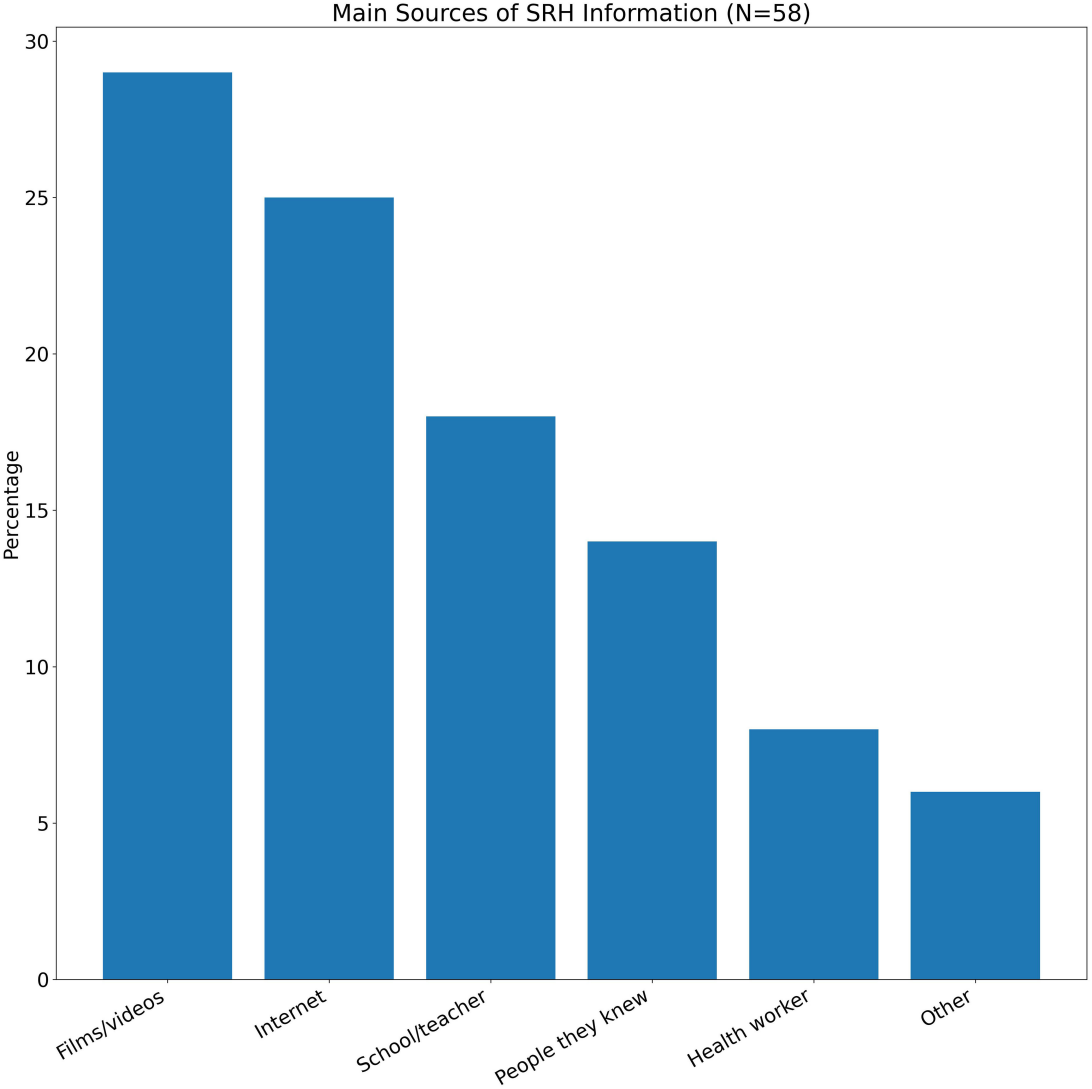
Sources of pornography and sexual knowledge (N=58). Films and videos were the main sources (29%), followed by the Internet (25%), school or teachers (18%), and peers (14%). Only 8% mentioned health workers. The reliance on non-educational sources emphasizes the lack of structured sexuality education.

About 29% of participants cited films or videos (implicitly pornography) as a primary source of sexual knowledge, 14% relied on people they knew, and a third were unsure (See **Figure 6**). Only a small proportion cited formal education or health providers. Pornography’s dominance as a knowledge source has far-reaching implications. Adolescents consuming unfiltered content—without media literacy or SRH context—may develop unrealistic expectations, internalize gender stereotypes, and engage in risky behaviors (56). Migrant youth, whose formal education is often interrupted and whose caregivers may avoid sexual topics, are especially susceptible to this trend.

### Access to information on puberty and menstruation

‘‘We talk between us. My friend explained things to me about periods and condoms. We help each other because no one else does.” — Female, 16, Gambia.

**Figure 7:**
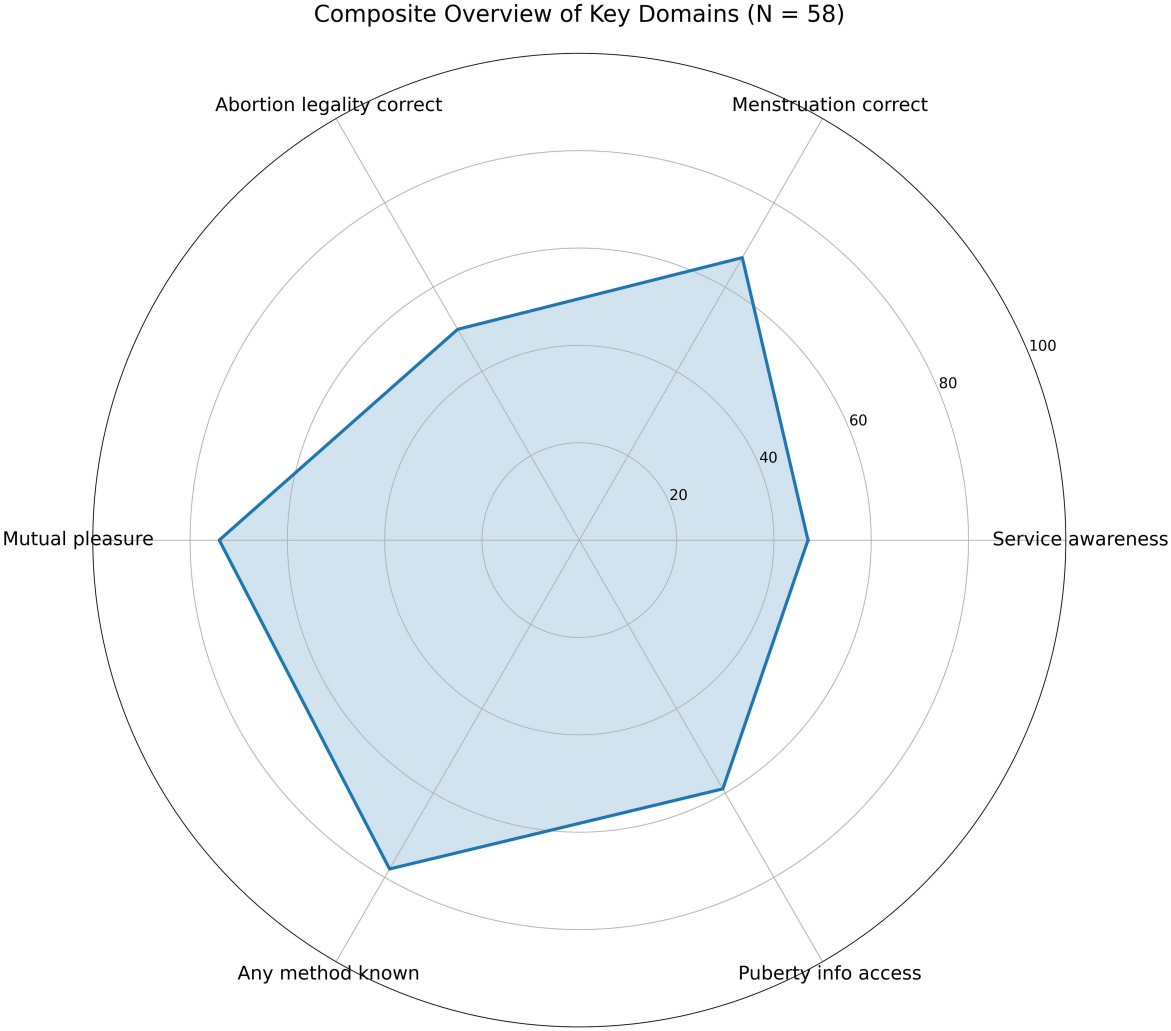
Access to information on puberty and menstruation (N=58). Fifty-nine percent said they could explain where to find SRH information, 14% could not, and 27% were unsure. These findings highlight persistent barriers to reliable SRH information, particularly for menstruating adolescents.

Only 59% of respondents stated they knew where to find information on puberty and menstruation, while 14% said they could not, and 27% were unsure (See **Figure 7**). Among menstruating adolescents, this lack of knowledge has physical, emotional, and logistical consequences—from shame and isolation to poor hygiene practices. Educational gaps here are not neutral: they reflect broader silences about female bodies in both host and origin cultures.

### Language preferences for SRH information

**Figure 8:**
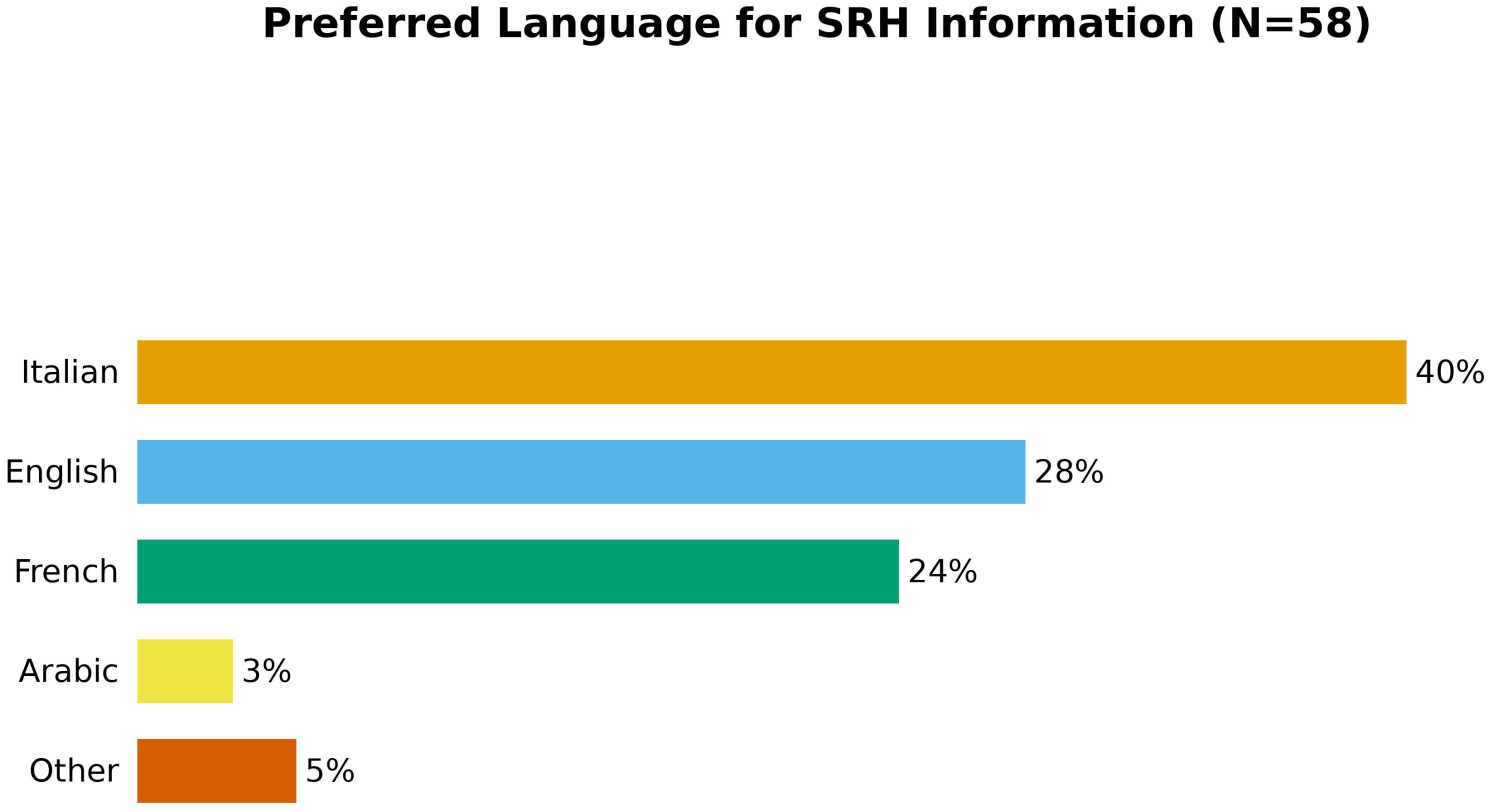
Language preferences for information (N=58). Forty percent preferred Italian, 28% English, 24% French, and 3% Arabic. Nearly 60% preferred a non-Italian language, underscoring the need for multilingual educational materials to reach diverse migrant youth communities.

While 40% of adolescents preferred Italian for SRH information, a significant 60% chose other languages: English (28%), French (24%), and Arabic (3%). These findings highlight the importance of multilingual health education materials and trusted translation services (See **Figure 8**). Language is not merely a communication tool—it shapes the comfort, trust, and psychological safety of healthcare interactions. When adolescents have to communicate in an unfamiliar language, especially about intimate or stigmatized topics, critical information may be lost or avoided altogether.

### Psychosocial resilience and adaptive strengths

“Even when things are hard, I try to help my younger cousin with what I know. We learn from each other. We don’t give up.” — Male, 16, Côte d’Ivoire

**Figure 9:**
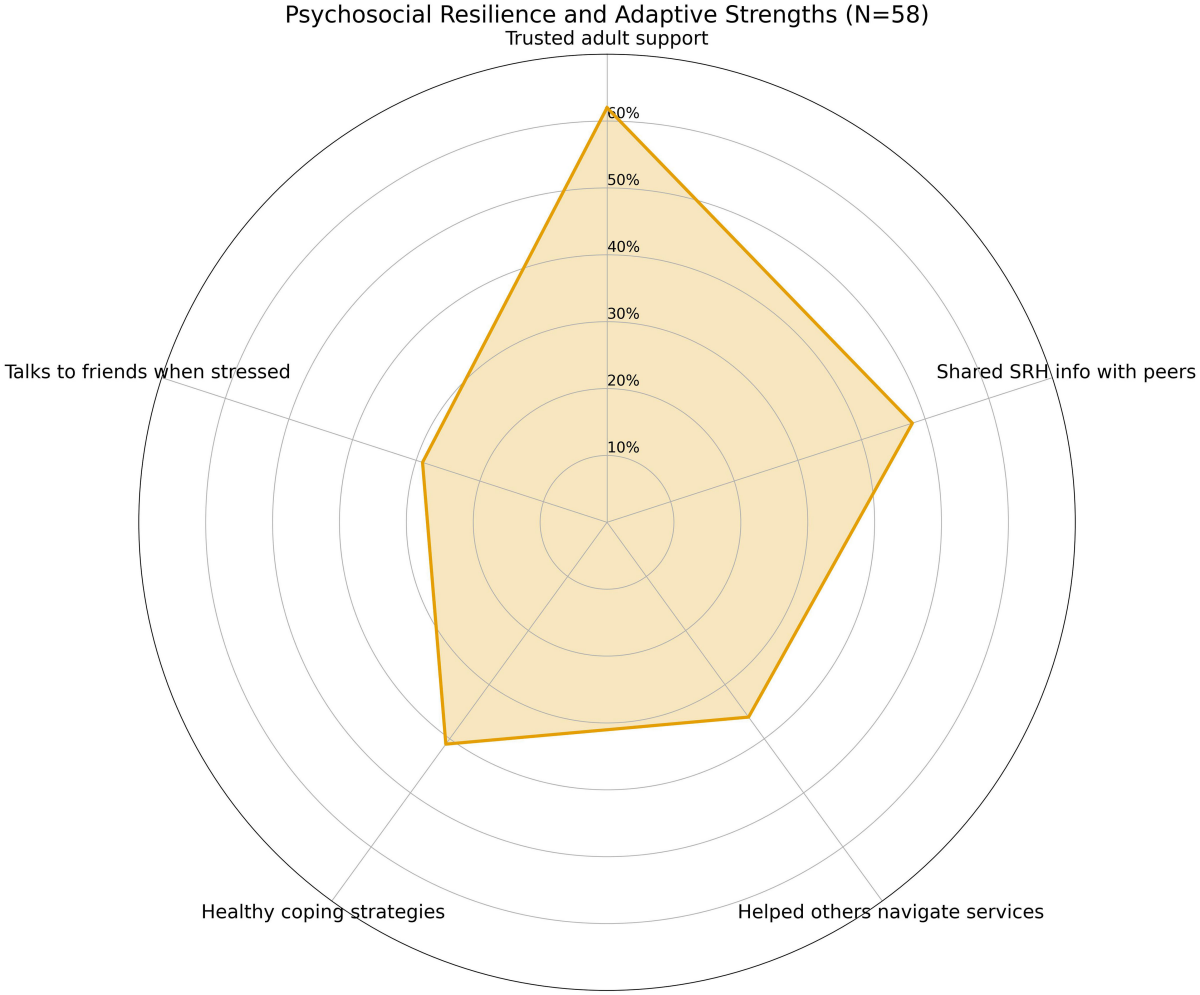
Psychosocial resilience and adaptive strengths (N = 58). Over 60% of participants reported having at least one trusted adult they could talk to about sensitive issues. Forty-eight percent had shared SRH information with peers, 36% had helped others navigate health-related services, 41% reported using healthy coping strategies, and 29% spoke with friends when stressed. These findings highlight informal systems of mutual aid, emotional regulation, and relational resilience among migrant adolescents.

Despite the vulnerabilities highlighted elsewhere, many participants showed signs of psychosocial resilience and adaptive functioning (See **Figure 9**). Over 60% reported having at least one trusted adult—such as a teacher, cultural mediator, or older sibling—to whom they could talk to about sensitive issues, including relationships or future plans. Additionally, 48% stated that they had shared SRH information with peers, and 36% reported helping someone else navigate health-related questions or services. These peer-oriented actions reflect informal caregiving roles and knowledge-sharing practices often overlooked in clinical assessments.

Further, when asked how they cope with stress, 41% identified non-destructive strategies such as physical activity, music, prayer, or journaling—while 29% cited speaking with a friend. These responses point to protective coping mechanisms that, while not replacing professional support, contribute to emotional regulation and social connection. These resilience indicators echo socio-ecological models which emphasize that resilience is not only an internal trait but a product of relational and contextual resources. Migrant adolescents may not always present with formal help-seeking behaviors, but their informal systems of mutual aid, emotional expression, and adaptive attitudes are vital strengths.

### Exposure to trauma and psychological strain

“Sometimes I see things in my head again—what happened before I came here. I don’t sleep well. But I can’t explain this to anyone.” — Female, 17, Pakistan.

**Figure 10:**
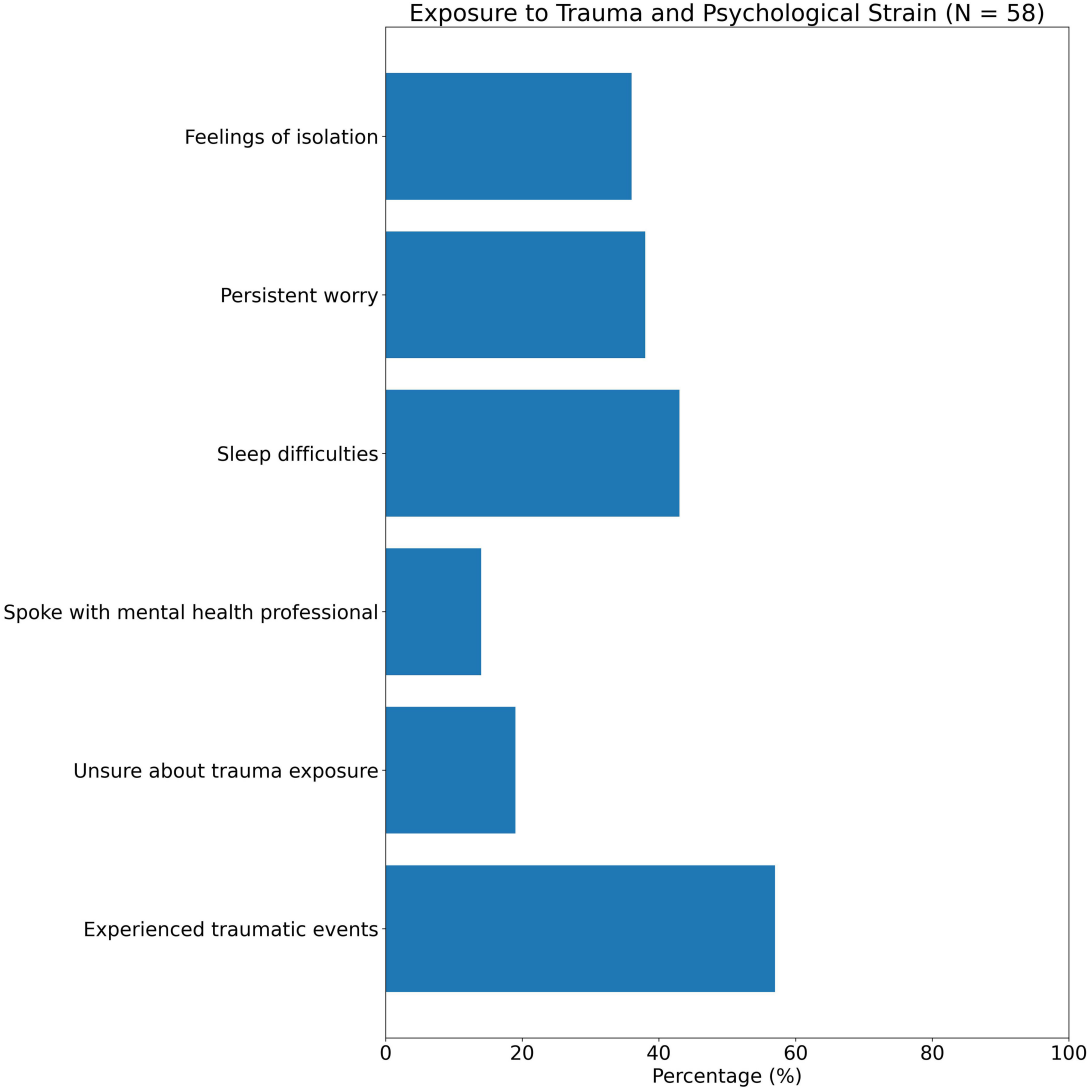
Exposure to trauma and psychological strain (N = 58). Fifty-seven percent reported experiencing traumatic events, with an additional 19% unsure. Only 14% had ever spoken with a mental health professional. Sleep difficulties (43%), persistent worry (38%), and feelings of isolation (36%) were common, illustrating a substantial burden of unaddressed psychological distress among migrant adolescents.

Trauma exposure was a common but often unspoken reality among participants. When asked whether they had experienced events that made them feel very afraid, unsafe, or helpless, 57% responded “yes,” with an additional 19% unsure (see **Figure 10**). This included witnessing violence during migration, experiencing abuse, or facing systemic exclusion in host contexts. Yet only 14% reported ever speaking with a mental health professional about these experiences.

Sleep difficulties (reported by 43%), persistent worry (38%), and feelings of isolation (36%) were frequent, although not always labeled as symptoms. Several participants associated distress with cultural displacement or fear of legal insecurity, rather than past trauma—highlighting the need for culturally nuanced approaches to psychological support. Notably, girls more often reported somatic expressions of distress (e.g., headaches, stomach pain), while boys were more likely to mention anger or detachment.

## Discussion

This exploratory survey of 58 migrant adolescents in Italy provides timely and critical insights into a rarely explored intersection: that of sexual and reproductive health (SRH), resilience, and adolescent mental health within migratory contexts. Although the sample size is limited and the design non-probabilistic, the study’s value lies not in its statistical generalizability but in its capacity to illuminate systemic mismatches, underexamined protective factors, and implications for psychiatric care. By situating adolescent SRH knowledge, beliefs, and attitudes within the broader social determinants of health, the study could help reposition migrant psychiatry as a field in urgent need of conceptual, clinical, and policy innovation.

At the heart of the findings is a revealing paradox. While many adolescents endorsed progressive attitudes—such as mutual pleasure, gender equity, and curiosity about sexual health—their knowledge base remained fragmented and shallow. Misconceptions about fertility and abortion legality were widespread; awareness of SRH services was inconsistent; and many adolescents identified pornography or peers as primary sources of information. This disconnect illustrates a form of cognitive-emotional dissonance: youth are open to engagement and attuned to equitable norms but are structurally constrained from translating those attitudes into safe and informed health behaviors.

This paradox is not confined to Italy. Across Europe, migrant adolescents are navigating overlapping systems—legal, educational, health, and cultural—that are often disjointed, exclusionary, or ill-prepared to accommodate their needs. Studies from Sweden, Germany, France, and the Netherlands confirm that while migrant youth are at elevated risk for psychosocial distress, their experiences are also shaped by interrupted education, xenophobia, stigma around SRH, and inadequate access to linguistically and culturally appropriate services (57–59). The compounding nature of these challenges—what some scholars refer to as “structural vulnerability”—means that mental health is never a purely clinical matter. It is embedded in the quality and accessibility of public systems. Yet, amid these systemic barriers, many adolescents in this study demonstrated indicators of resilience and adaptability. High endorsement of gender-equal norms and mutual respect in relationships suggests a receptiveness to positive sexual ethics. The expressed desire to learn more about SRH, coupled with active engagement in informal learning channels—even if imperfect—underscores an agency that is often overlooked in clinical discourses that focus narrowly on trauma and risk.

### Positioning the findings within broader scholarly context

The results of this exploratory study both confirm and extend existing scholarship on migrant adolescent health in Europe. Consistent with previous findings, the data reaffirm that migrant youth often experience significant gaps in sexual and reproductive health (SRH) literacy, limited awareness of legal rights (such as abortion access), and difficulties navigating fragmented service systems (21, 24, 28). However, the study also contributes novel insights by foregrounding psychosocial resilience and progressive gender norms—features that are frequently underrepresented in psychiatric literature. Unlike traditional deficit-based approaches, these findings suggest that many adolescents exhibit adaptive strategies, emotional regulation, and peer-driven knowledge sharing despite structural vulnerabilities. In this sense, the study expands resilience literature by illustrating how informal support networks and self-efficacy can function as protective mechanisms in migratory contexts (34, 36, 38). Moreover, the observed confusion around SRH services and legal rights challenges assumptions that residence in a host country automatically results in health system integration or knowledge acquisition (18, 19, 23).

### Humanistic and interdisciplinary framing

At the heart of this study lies an ethical commitment to a humanistic and rights-based vision of care—one that challenges the traditional confines of psychiatric knowledge production when applied to marginalized, often invisibilized, migrant youth. Mainstream psychiatric models frequently overlook the lived realities of legal precarity, structural violence, and non-recognition of personhood that shape the trajectories of displaced adolescents. These are not peripheral issues; they are central to how risk, trauma, and even so-called “risky” behaviors (such as transactional sex or border-crossing strategies) are enacted and experienced. For many adolescents in this study, such behaviors may represent forms of resilience, autonomy, or survival within hostile or exclusionary systems. Critically, the focus on sexual and reproductive health (SRH) risks should not eclipse the broader systemic failures—including inadequate asylum systems, detention practices, and exclusion from formal schooling—that drive vulnerability.

### Interpreting knowledge gaps and misinformation

Limited awareness of contraceptive options—particularly long-acting reversible contraceptives (LARCs) and emergency contraception—was evident. Most participants could name male condoms, but only a few recognized the pill or injectable methods, and knowledge of IUDs was negligible. Similar patterns have been documented in studies of migrant youth across Europe, where barrier methods tend to be overrepresented and female-controlled options underrecognized (60).

Misconceptions about fertility during menstruation and legal confusion around abortion further complicate adolescents’ ability to make informed choices. This legal literacy gap is not benign: adolescents who are uncertain about the legality of abortion are less likely to seek timely care, more vulnerable to misinformation, and at risk of internalizing stigma. Studies in Italy and other Southern European countries confirm that both migrant and host country-born youth often lack accurate legal knowledge about SRH rights, contributing to fear, silence, and delay in help-seeking (61).

### Structural barriers and service awareness

Less than half of participants reported knowing where to access SRH services. This is consistent with findings from the European Youth Health Survey, which show that migrants—especially unaccompanied minors—face “triple barriers”: informational deficits, systemic fragmentation, and sociocultural mistrust of institutions (62). Without basic awareness of available services, even well-intentioned or well-designed interventions fail to reach their audience. This gap has direct psychiatric implications. Adolescents who lack access to trusted SRH information and services are at greater risk of experiencing unplanned pregnancies, STIs, and psychological distress—including anxiety, shame, and guilt. In practice, this may result in late presentation to mental health services, often during crises rather than early intervention windows.

### Progressive norms as protective factors

One of the most encouraging findings was the endorsement of progressive attitudes toward sexual relationships: over 70% of participants agreed that both partners should enjoy intimacy. This signals a normative shift toward mutuality and respect—values associated with gender equality, communication, and emotional wellbeing.

From a resilience framework, these beliefs function as psychosocial assets. Numerous studies show that adolescents who endorse egalitarian gender norms are more likely to report healthy relationships, lower rates of coercion, and better mental health outcomes (63).

### The role of informal sources: pornography as surrogate curriculum

A significant minority of adolescents cited pornography as a main source of sexual knowledge, reflecting broader global patterns in contexts where formal SRH education is absent. While adolescents may turn to pornography out of curiosity or necessity, such content often portrays unrealistic, stereotyped, or violent representations of sexuality, which can shape distorted expectations and emotional distress—especially in the absence of critical media literacy. This reliance is particularly concerning for migrant adolescents, who may face additional taboos, lack trusted adults, or experience language barriers in formal settings.

### Beyond the pornography risk lens

While the data suggest that pornography is a common source of sexual knowledge for many participants, framing this solely as risk obscures deeper, more pressing vulnerabilities. In the context of precarious migration, young people often face systemic exclusion from formal education, health systems, and legal protections. These structural deficits, more than any exposure to online sexual content, shape adolescent experiences of risk and agency. Behaviors that are often labeled as “risky”—such as engaging in transactional sex, forming age-discrepant relationships, or withholding disclosure of sexual activity—may be strategic responses to material insecurity, legal invisibility, or the aspiration to migrate onwards. These are not merely psychological phenomena, but socio-political ones.

### Language as a structural determinant of access

Over half of participants preferred SRH information in a language other than Italian—most commonly English, French, or Arabic. This is a potent reminder that language is not a superficial communication issue but a structural determinant of health. When health information is available in languages youth understand, their access to services, comprehension of rights, and trust in institutions augment.

Multilingualism should therefore be considered a non-negotiable element of youth services. Cultural mediators, translated materials, and bilingual peer educators are not “extras”—they are prerequisites for equity.

### Consent and guardianship: legal ambiguities and barriers to care

Migrant adolescents in Italy—particularly unaccompanied minors—often face complex legal arrangements regarding guardianship and consent. While Italian law affirms adolescents’ right to confidential healthcare, including access to sexual and reproductive health services, in practice, the interpretation of consent under guardianship is inconsistent. Some healthcare providers may require adult accompaniment or explicit permission from legal guardians before offering contraception, abortion counseling, or even SRH information. This creates significant access barriers, particularly for those in shelters or informal living arrangements. The resulting confusion—among adolescents, guardians, and providers alike—can delay care, deter disclosure, and amplify stigma. Clarity and training on consent frameworks are urgently needed, ensuring that all adolescents, regardless of status, can exercise their health rights safely and autonomously.

### Sexual violence and SRH confusion

An often-overlooked dimension of SRH vulnerability among migrant adolescents is exposure to sexual violence and exploitation. For many, displacement and precarious migration pathways increase the risk of coercion, transactional sex, or abuse within shelters, communities, or even bureaucratic systems. These experiences are rarely disclosed due to stigma, fear of legal repercussions, or mistrust of authorities. When compounded by limited SRH literacy and confusion about consent, contraception, and legal rights, the consequences can be severe, from unintended pregnancies and untreated STIs to compounding trauma and psychiatric distress. The absence of clear, youth-friendly, and rights-based information on sexual health leaves survivors without the tools to process their experiences or seek support. In such cases, the boundary between psychiatric care and SRH education dissolves: addressing trauma requires not only psychological support, but also accessible knowledge about the body, consent, and care-seeking pathways.

### Aligning local evidence with global frameworks

The patterns uncovered in this study resonate with and are reinforced by international evidence and policy guidance. The World Health Organization’s Global Accelerated Action for the Health of Adolescents (AA-HA)! framework underscores that adolescent wellbeing depends on access to SRH, mental health, and social protection systems that are developmentally appropriate, youth-centered, and culturally responsive (64). For migrant adolescents, this means services should be tailored to their legal, linguistic, and psychosocial contexts—not merely translated from mainstream models.

UNESCO’s International Technical Guidance on Sexuality Education (ITGSE) likewise identifies comprehensive sexuality education (CSE) as a foundational component of adolescent development. Yet, Italy remains one of the few European countries without a national mandate for school-based CSE. The result is a patchwork of delivery dependent on local actors, often leaving migrant adolescents—whose schooling is already disrupted—excluded from essential knowledge. This reinforces the study’s finding that adolescents rely on peers, media, and guesswork to fill information gaps (65).

Adolescents who lack reliable SRH information are more likely to internalize shame, fear, or confusion—particularly if their questions are met with silence or punishment in their family or cultural context. Integrating CSE into mental health promotion—via community settings, schools, and clinical interactions—can help bridge this gap.

Resilience theory, as developed by scholars like Michael Ungar (66), further helps contextualize these findings. Resilience is not simply about individual traits; it is about the interaction between personal agency and access to supportive resources. Migrant adolescents may possess adaptive capacity, but it should be scaffolded by systems that recognize and amplify their strengths (67). This includes not only SRH literacy but also emotional support, legal information, and relational safety.

Similarly, trauma-informed care (TIC) offers a critical orientation. Adolescents affected by displacement, legal insecurity, and systemic exclusion experience complex forms of trauma that extend beyond traditional diagnostic categories. TIC emphasizes the need for safety, trust, empowerment, and cultural humility in service provision. Emerging programs in Belgium and Canada, which co-locate SRH, legal aid, and mental health support, have shown success in increasing adolescent engagement and reducing crisis-level presentations (68).

The dual frameworks of resilience theory and trauma-informed care (TIC) offer powerful, complementary lenses through which to interpret the findings of this study. Resilience theory posits that young people possess inherent and context-dependent capacities to adapt and even thrive under adversity, particularly when supported by enabling environments. This process is not solely individual but fundamentally ecological, emerging through interaction with family, peers, institutions, and broader sociocultural systems. Migrant adolescents, despite facing disrupted schooling, linguistic marginalization, and precarious legal status, often exhibit proactive efforts to seek knowledge, express autonomy, and align with gender-equitable values. These are not merely coping strategies but expressions of developmental strength and psychosocial potential that psychiatric systems can scaffold.

Trauma-informed care, by contrast, begins from the premise that service delivery should be grounded in an understanding of the prevalence and impact of trauma, particularly among populations affected by displacement, exclusion, and systemic violence. TIC emphasizes safety, trustworthiness, empowerment, collaboration, and cultural humility as guiding principles. For migrant adolescents, such care requires more than symptom assessment or clinical intervention; it calls for services that acknowledge the structural conditions of legal uncertainty, institutional neglect, and social invisibility. Rather than pathologizing responses to these conditions, trauma-informed approaches validate them and seek to reduce retraumatization within health systems themselves.

In combination, resilience and TIC frameworks challenge the assumption that vulnerability is the only defining characteristic of migrant adolescents. Instead, these approaches allow for the coexistence of strength and suffering, agency and adversity. In the current study, adolescents’ endorsement of mutual pleasure in relationships and their desire for SRH knowledge suggest latent psychosocial assets. However, these strengths may remain untapped—or even suppressed—when systems fail to offer clear, culturally competent information or when adolescents encounter discrimination or silence in clinical encounters. The widespread lack of service awareness, as identified in the survey, may reflect not only gaps in dissemination but also prior negative or invalidating experiences within institutional settings.

Integrating resilience and trauma-informed frameworks into psychiatric practice therefore involves not only clinical adjustments, but systemic and epistemic shifts. These include expanding assessment protocols to include questions on SRH literacy, service access, and identity-related stressors; incorporating multilingual and culturally congruent mediators into care pathways; and reframing psychiatry’s role from diagnosing individual pathology to co-producing psychosocial wellbeing with adolescents. This reframing aligns with global calls for adolescent-responsive systems and reflects a more just, context-sensitive model of mental health support for migrant youth.

Programs like Boys on the Move (BotM), developed by UNFPA and UNICEF, could maybe help in operationalizing these principles by delivering SRH literacy, coping strategies, and life-skills education in culturally relevant formats. Evidence from Italy shows that BotM could possibly improve knowledge, reduce stigma, and foster peer support. Psychiatry could partner with such programs, extending their reach and offering a continuum of care from community education to clinical services (69).

Another example of accessible, multilingual sexual health information is the Zanzu platform, developed by the German Federal Centre for Health Education (BZgA) in collaboration with Sensoa, Belgium’s Flemish expert center for sexual health. Zanzu provides culturally sensitive SRH information in over 13 languages, including Arabic, Tigrinya, Farsi, and French, making it relevant for newly arrived migrants and refugees. The platform covers topics such as contraception, consent, pregnancy, and rights in simple, visual formats designed for low-literacy audiences. Although originally developed for Germany and Belgium, Zanzu represents a scalable model that could be adapted in Italy to address the critical language-related barriers reported by our participants. Integrating such tools into reception centers, shelters, schools, and psychiatric care settings could probably enhance the accessibility and cultural relevance of SRH communication.

### Migration as structural condition, not demographic variable

Migration in this study should not be understood merely as a demographic characteristic, but as a structural and political condition that organizes access to care, visibility, and personhood. Many of the adolescents engaged in this research are not simply “migrants” in a legalistic sense but are navigating overlapping regimes of exclusion—including denied asylum, age-disputed status, temporary reception, and systemic racialization. These exclusions shape not only access to SRH services but also the very meanings of risk, vulnerability, and agency. As Giordano argues in Migrants in Translation (64), young migrants in Italy are routinely caught in translational gaps between institutional care systems and their lived experiences, where legal and psychiatric frameworks fail to recognize their subjectivity or the complexity of their survival strategies. Migration trauma, thus, cannot be reduced to pre-migration events or border crossings—it extends into the chronic uncertainty of daily life in the receiving country, where sexuality, consent, and care become entangled with bureaucratic and carceral logics.

### Limitations

This study, while offering valuable insights into the intersection of migrant adolescent mental health and SRH knowledge, has several limitations that warrant consideration. First, the sample size was modest (n = 58) and recruited through convenience sampling in specific reception centers, which may limit the generalizability of findings to broader migrant youth populations in Italy or across Europe. Second, the self-report nature of the survey, particularly regarding sensitive topics such as contraception, abortion, and sexual experience, may have introduced social desirability bias or recall inaccuracies. Although efforts were made to ensure confidentiality, participants may have withheld or modified responses due to fear, stigma, or mistrust. Third, the survey was administered in simplified Italian or through translation, which, despite cultural mediators’ involvement, may have affected comprehension or nuance in interpretation—especially among participants with limited literacy. Fourth, the absence of qualitative data or direct quotations restricts the ability to capture the depth and complexity of adolescent lived experience. Finally, the cross-sectional design limits the capacity to draw conclusions about causality or longitudinal changes in knowledge, attitudes, or service access over time.

Future research should seek to address these limitations by using larger, more representative samples, employing mixed-methods approaches to enrich interpretation, and exploring longitudinal and intervention-based designs. Including adolescents as co-researchers could further enhance cultural relevance, trust, and depth of analysis.

### Reflexivity

As researchers working across global health and youth migration, we are mindful of the ways our positionalities—academic, institutional, cultural—have shaped the design, interpretation, and presentation of this study. While the survey was conducted with the support of cultural mediators and youth workers to reduce power imbalances and enhance trust, we recognize that all knowledge production is situated. The adolescents’ responses may have been influenced by perceived social desirability, language comfort, or assumptions about authority. We aimed to mitigate this through culturally sensitive phrasing, informed consent processes, and voluntary participation. Still, our analysis inevitably reflects the interpretive frameworks and disciplinary lenses we bring to the data. We emphasize that this work is not a definitive account of migrant adolescent experience, but a contribution to an ongoing, participatory dialogue that should center youth voices more fully in future research and practice.

## Conclusion: toward a holistic and resilience-oriented migrant psychiatry

The implications of this study are both clinical and systemic. First and foremost, psychiatry should expand its remit. A narrow focus on trauma and symptomatology is insufficient. Instead, adolescent mental health care—especially in migration-affected contexts—should engage with SRH, resilience, and rights as integral to wellbeing.

Clinicians are advised to:

- Integrate SRH knowledge checks and psychoeducation into psychiatric assessments;
- Collaborate with cultural mediators to overcome language and trust barriers;
- Recognize progressive attitudes as protective, not naive;
- Address media literacy, including critical discussions of pornography.

Systemically, reforms are needed at multiple levels:

- Policy: Italy and similar countries should mandate culturally adapted, age- and developmental stage-appropriate CSE in schools and shelters, ensuring universal access.
- Service design: Youth hubs should co-locate mental health, SRH, and legal services to reduce fragmentation.
- Research: Future studies should use longitudinal, mixed-method designs and include adolescents as co-researchers to capture lived realities and enhance relevance.
- Monitoring: Routine data collection on migrant adolescents’ SRH and mental health—disaggregated by gender, status, and origin—is essential to informed policymaking.

Finally, youth participation is not optional. Migrant adolescents should be recognized not as passive beneficiaries but as active agents whose insights can and should shape services. Participatory design, adolescent advisory boards, and peer-led interventions offer not only ethical engagement but also practical effectiveness.

In a time marked by rising nationalism, misinformation, and austerity, investing in migrant adolescent wellbeing is more than a health imperative—it is a social justice issue. Psychiatry, long reticent to engage with SRH, now has an opportunity to lead. By bridging the gaps between knowledge, rights, and care, it can transform from a siloed discipline into a field of solidarity, dignity, and flourishing.

## Data Availability

The raw data supporting the conclusions of this article will be made available by the authors, without undue reservation.

## References

[1] United Nations Population Fund (UNFPA) . Sexual and Reproductive Health and Rights: An Essential Element of Universal Health Coverage. New York: UNFPA (2019).

[2] WHO . Adolescent Pregnancy. Geneva: World Health Organization (2020).

[3] UNFPA . My Body is My Own: Claiming the Right to Autonomy and Self-Determination. In: State of World Population. UNFPA, New York (2021).

[4] WHO . Global Accelerated Action for the Health of Adolescents (AA-HA)!: Guidance to Support Country Implementation. Geneva: World Health Organization (2017).

[5] United Nations Human Rights Council . Report of the Special Rapporteur on the Right of Everyone to the Enjoyment of the Highest Attainable Standard of Physical and Mental Health. Geneva: UNHRC (2019).

[6] Deogan C Ferguson BJ Stenberg K . Resource needs for adolescent friendly health services: estimates for 74 low- and middle-income countries. PLoS One. (2012) 7:e51420. doi: 10.1371/journal.pone.0051420, PMID: 23300548 PMC3531400

[7] Bearinger LH Sieving RE Ferguson J Sharma V . Global perspectives on the sexual and reproductive health of adolescents: patterns, prevention, and potential. Lancet. (2007) 369:1220–31. doi: 10.1016/S0140-6736(07)60367-5, PMID: 17416266

[8] Chandra-Mouli V Lane C Wong S . What does not work in adolescent sexual and reproductive health: a review of evidence on interventions commonly accepted as best practices. Glob Health Sci Pract. (2015) 3:333–40. doi: 10.9745/GHSP-D-15-00126, PMID: 26374795 PMC4570008

[9] Tol WA Song S Jordans MJ . Annual Research Review: Resilience and mental health in children and adolescents living in areas of armed conflict–a systematic review of findings in low-and middle-income countries. J Child Psychol Psychiatry. (2013) 54:445–60. doi: 10.1111/jcpp.12053, PMID: 23414226

[10] Masten AS . Ordinary magic: Resilience in development. New York: Guilford Press (2014).

[11] Bhugra D Till A Sartorius N . What is a psychiatric disorder? A proposed model. World Psychiatry. (2013) 12:308–17.

[12] Tribe R . Bridging the gap or damming the flow? Some observations on using interpreters/bilingual workers when working with refugee children, young people and families. Int J Migration Health Soc Care. Oxford: Oxford University Press. (2014) 10:52–60. doi: 10.1348/000711299160130, PMID: 10616138

[13] Kirmayer LJ . Cultural psychiatry in historical perspective. In: Bhugra D Bhui K , editors. Textbook of Cultural Psychiatry. Cambridge: Cambridge University Press (2007). p. 3–19.

[14] Ungar M . Multisystemic resilience: Adaptation and transformation in contexts of change. Oxford: Oxford University Press (2021).

[15] Panter-Brick C Leckman JF Eggerman M . Understanding culture, resilience, and mental health: The production of hope. In: Ungar M , editor. The social ecology of resilience. Springer, New York (2012). p. 369–86.

[16] Theron L . Resilience research with LGBTQ+ youth: The importance of context. In: Ungar M , editor. Multisystemic Resilience. Oxford University Press, New York (2021). p. 233–48.

[17] Betancourt TS Khan KT . The mental health of children affected by armed conflict: protective processes and pathways to resilience. Int Rev Psychiatry. (2008) 20:317–28. doi: 10.1080/09540260802090363, PMID: 18569183 PMC2613765

[18] Fazel M Reed RV Panter-Brick C Stein A . Mental health of displaced and refugee children resettled in high-income countries: risk and protective factors. Lancet. (2012) 379:266–82. doi: 10.1016/S0140-6736(11)60051-2, PMID: 21835459

[19] Save the Children . Still at Risk: The Dangers of Living on the Move for Refugee and Migrant Children Arriving in Europe. London: Save the Children (2020).

[20] Creswell JW Plano Clark VL . Designing and Conducting Mixed Methods Research. 3rd ed. Thousand Oaks, CA: SAGE Publications (2017).

[21] Morse JM . Critical analysis of strategies for determining rigor in qualitative inquiry. Qual Health Res. (2015) 25:1212–22. doi: 10.1177/1049732315588501, PMID: 26184336

[22] Patton MQ . Qualitative Research and Evaluation Methods. 4th ed. Thousand Oaks, CA: SAGE Publications (2015).

[23] UNFPA UNICEF . Boys on the Move: A Life Skills Curriculum for Unaccompanied and Separated Migrant Boys in Transit. New York: UNFPA and UNICEF (2019).

[24] UNFPA UNICEF . 12 Questions and Answers on Sexual and Reproductive Health and Rights (SRHR). New York: UNFPA and UNICEF (2019).

[25] Marston C Renedo A . Understanding and measuring youth empowerment processes: a co-creation approach in the UK. Glob Health Action. (2013) 6:21102. doi: 10.1186/s12889-025-24095-z, PMID: 40885906 PMC12398034

[26] Chandra-Mouli V Akwara E Engel D Plessons M . Progress in adolescent sexual and reproductive health and rights globally between 1990 and 2016. J Adolesc Health. (2021) 68:S1–3. doi: 10.1080/26410397.2020.1741495, PMID: 32254004 PMC7888102

[27] Kennedy EC Bulu S Harris J Humphreys D Malverus J Gray NJ . These issues aren’t talked about at home”: a qualitative study of the sexual and reproductive health information preferences of adolescents in Vanuatu. BMC Public Health. (2014) 14:770. doi: 10.1186/1471-2458-14-770, PMID: 25073619 PMC4133612

[28] Dick B Ferguson BJ Chandra-Mouli V Brabin L Chatterjee S Ross DA . Review of the evidence for interventions to increase young people’s use of health services in developing countries. World Health Organ Tech Rep Ser. (2006) 938:151–204. 16921920

[29] Tylee A Haller DM Graham T Churchill R Sanci LA . Youth-friendly primary-care services: how are we doing and what more needs to be done? Lancet. (2007) 369:1565–73. doi: 10.1016/S0140-6736(07)60371-7, PMID: 17482988

[30] Graham A Powell M Taylor N Anderson D Fitzgerald R . Ethical Research Involving Children. Florence: UNICEF Office of Research-Innocenti (2013).

[31] UNHCR . Desperate Journeys: Refugee and Migrant Children arriving in Europe and how we can support them. Geneva: United Nations High Commissioner for Refugees (2020).

[32] Lindert J Carta MG Schäfer I Mollica RF . Refugees mental health–a public mental health challenge. Eur J Public Health. (2016) 26:374–5. doi: 10.1093/eurpub/ckw010, PMID: 27053728

[33] Bhugra D Gupta S Bhui K Craig TK Dogra N Ingleby JD . WPA guidance on mental health and mental health care in migrants. In Mental Health, Mental Illness and Migration. Singapore: Springer Singapore (2021) pp. 613–630.

[34] van Os J Guloksuz S . A critique of the “ultra-high risk” and “transition” paradigm. World Psychiatry. (2017) 16:200–6. doi: 10.1002/wps.20423, PMID: 28498576 PMC5428198

[35] Ungar M . Multisystemic resilience: Adaptation and transformation in contexts of change. Oxford: Oxford University Press (2021).

[36] Theron L Liebenberg L Ungar M . Youth resilience and the science of hope: A global perspective. London: Routledge (2015).

[37] Masten AS . Global perspectives on resilience in children and youth. Child Dev. (2014) 85:6–20. doi: 10.1111/cdev.12205, PMID: 24341286

[38] Kirmayer LJ Narasiah L Munoz M Rashid M Ryder AG Guzder J . Common mental health problems in immigrants and refugees: general approach in primary care. CMAJ. (2011) 183:E959–67. doi: 10.1503/cmaj.090292, PMID: 20603342 PMC3168672

[39] Braun V Clarke V . Successful qualitative research: A practical guide for beginners. London: SAGE (2013).

[40] Silove D Ventevogel P Rees S . The contemporary refugee crisis: an overview of mental health challenges. World Psychiatry. (2017) 16:130–9. doi: 10.1002/wps.20438, PMID: 28498581 PMC5428192

[41] Betancourt TS Meyers-Ohki SE Charrow A Hansen N . Annual research review: mental health and resilience in HIV/AIDS-affected children – a review of the literature and recommendations for future research. J Child Psychol Psychiatry. (2013) 54:423–44. doi: 10.1111/j.1469-7610.2012.02613.x, PMID: 22943414 PMC3656822

[42] Knaul FM Bhadelia A Atun R Frenk J . Achieving effective universal health coverage and diagonal approaches to care for chronic illnesses. Health Aff. (2015) 34:1514–22. doi: 10.1377/hlthaff.2015.0514, PMID: 26355053

[43] Marquez PV Farrington JL . The challenge of non-communicable diseases and road traffic injuries in Sub-Saharan Africa: An overview. Washington DC: World Bank (2013).

[44] Council of Europe . Youth work in the context of migration and asylum: reflections and guidelines. Strasbourg: Council of Europe Publishing (2017).

[45] European Commission . Action Plan on the integration of third-country nationals. Brussels: European Union (2016).

[46] de Graaff P Schouten S . Comprehensive sexuality education for young people in migrant communities. Sex Educ. (2021) 21:489–502. doi: 10.1007/978-3-031-40858-8_126-1

[47] UNFPA UNICEF . Boys on the Move: A life skills curriculum for unaccompanied and separated migrant boys in Europe. New York: United Nations Population Fund and United Nations Children’s Fund (2019).

[48] Glick Schiller N Basch L Blanc-Szanton C . From immigrant to transmigrant: Theorizing transnational migration. Anthropol Q. (1995) 68:48–63. doi: 10.2307/3317464, PMID: 39964225

[49] Betancourt TS Williams TP Kellner SE Gebre-Medhin J Hann K Kayiteshonga Y . Interventions for children affected by war: An ecological perspective on psychosocial support and mental health care. Harv Rev Psychiatry. (2011) 19:22–33. doi: 10.1097/HRP.0b013e318283bf8f, PMID: 23656831 PMC4098699

[50] Silove D . The ADAPT model: A conceptual framework for mental health and psychosocial programming in post-conflict settings. Intervention. (2013) 11:237–48. doi: 10.1097/WTF.0000000000000005, PMID: 41710530

[51] Ventevogel P van Ommeren M Schilperoord M Saxena S . Improving mental health care in humanitarian emergencies. Bull World Health Organ. (2015) 93:666–666A. doi: 10.2471/BLT.15.156919, PMID: 26600604 PMC4645443

[52] Graham A Powell MA Taylor N Anderson D Fitzgerald R . Ethical research involving children. Florence: UNICEF Office of Research–Innocenti (2013).

[53] Save the Children . Little Invisible Slaves: Voices of child victims of trafficking in Italy. Rome: Save the Children Italy (2020).

[54] United Nations . Convention on the Rights of the Child. Geneva: United Nations (1989).

[55] Creswell JW Plano Clark VL . Designing and conducting mixed methods research. 2nd ed. Thousand Oaks: SAGE Publications (2017).

[56] Morse JM . Approaches to qualitative-quantitative methodological triangulation. Nurs Res. (1991) 40:120–3. doi: 10.1097/00006199-199103000-00014, PMID: 2003072

[57] Theron L . Resilience of sub-Saharan adolescents: Theoretical perspectives, research findings, and pathways to resilience-promoting interventions. Child Adolesc Psychiatr Clin N Am. (2016) 25:333–44. doi: 10.1177/1363461520938916, PMID: 32723159

[58] Ungar M . Practitioner review: Diagnosing childhood resilience – a systemic approach to the diagnosis of adaptation in adverse social and physical ecologies. J Child Psychol Psychiatry. (2015) 56:4–17. doi: 10.1111/jcpp.12306, PMID: 25130046

[59] Rotheram-Borus MJ Swendeman D Becker KD . Adolescent health and wellness: Health, resilience, and youth development. Annu Rev Clin Psychol. (2014) 10:441–65. doi: 10.1080/15374416.2013.836453, PMID: 24079747 PMC3954876

[60] Council of Europe . Standards for youth policy. Strasbourg: Council of Europe Publishing (2015).

[61] Patton MQ . Qualitative research and evaluation methods. 3rd ed. Thousand Oaks: SAGE Publications (2015).

[62] Tol WA Rees S Silove D . Broadening the scope of epidemiology in conflict-affected settings: Opportunities for mental health prevention and promotion. Epidemiol Psychiatr Sci. (2013) 22:197–203. doi: 10.1017/S2045796013000188, PMID: 23941725 PMC8367339

[63] Graham A Fitzgerald R . Children’s participation in research: Some possibilities and constraints in the current Australian research environment. J Sociol. (2010) 46:133–47. doi: 10.1177/1440783309355065, PMID: 41783398

[64] Giordano C . Migrants in Translation: Caring and the Logics of Difference in Contemporary ITALY. Berkeley: University of California Press (2014).

[65] Bundeszentrale für gesundheitliche Aufklärung (BZgA), Sensoa. Zanzu – My body in words and images. Cologne: BZgA. Available online at: https://www.zanzu.de/en (Accessed November 15, 2025).

[66] Theron L Ungar M . Adolescent resilience in disadvantaged environments. In: Selin H Davey G , editors. Child and adolescent development: An international perspective. Springer, Dordrecht (2016). p. 289–304.

[67] Summerfield D . Cross-cultural perspectives on the medicalization of human suffering. BMJ. (2001) 322:1357–60. doi: 10.1002/9780470713570.ch12, PMID: 41784088

[68] Mills C Fernando S . Globalising mental health or pathologising the global south? Disability Global South. (2014) 1:188–216. Available online at: https://disabilityglobalsouth.files.wordpress.com/2012/06/dgs-01-02-00.pdf (Accessed September 15, 2025)

[69] Das V . Affliction: Health, disease, poverty. New York: Fordham University Press (2015).

