# Correction: Promoting resilience and sexual and reproductive health among adolescent migrants: a comprehensive approach

**DOI:** 10.3389/fpsyt.2026.1818321

**Published:** 2026-03-09

**Authors:** Alexios Georgalis, Robert Thomson

**Affiliations:** 1 Department of Psychology, National and Kapodistrian University of Athens, Athens, Greece; 2 Department of Social Work and Psychology, University of Gävle, Gävle, Sweden

**Keywords:** adolescent resilience, Italy, life-skills education, migrant psychiatry, psychosocial support, sexual and reproductive health, trauma-informed care, unaccompanied minors

An incorrect **Funding statement** was provided. The original article incorrectly stated that “the Geneva Health Forum fully covered the Article Processing Charge”. This should say “partly supported”. The correct **Funding statement** reads:

“The author(s) declared that financial support was received for this work and/or its publication. This study was supported by UNICEF Italy, in collaboration with UNFPA, as part of broader programming on adolescent health, protection, and inclusion in migration contexts. Funding covered the design, implementation, and ethical oversight of the study, including support for data collection tools and training. The funders had no role in the data analysis, interpretation of findings, or drafting of the manuscript. The Article Processing Charge for this manuscript was partly supported by the Geneva Health Forum. The authors would like to explicitly acknowledge and sincerely appreciate the Geneva Health Forum’s support, which made the open-access publication of this work possible. No additional external funding or grant numbers apply. Final decisions regarding publication rest solely with the authors”.

The original version of this article has been updated.

